# Monoclonal Antibodies in Relapsed-Refractory Multiple Myeloma

**DOI:** 10.3390/ph18020145

**Published:** 2025-01-22

**Authors:** Ilaria Sorgiovanni, Maria Livia Del Giudice, Sara Galimberti, Gabriele Buda

**Affiliations:** Hematology Division, University of Pisa, via Roma, 67, 56126 Pisa, Italy; ilaria.sorgiovanni01@gmail.com (I.S.); sara.galimberti@unipi.it (S.G.); gabriele.buda@unipi.it (G.B.)

**Keywords:** monoclonal antibodies, immunotherapy, multiple myeloma

## Abstract

Multiple myeloma is a malignant hematological tumor characterized by the proliferation of clonal plasma cells in the bone marrow causing organ damage. Despite improved survival rates due to the increasing availability of therapeutic options in recent decades, it remains an incurable disease, with most patients ultimately relapsing. Consequently, relapsed/refractory multiple myeloma disease (RRMM) has become a treatment priority. Immunotherapy is the backbone of treatment in RRMM, starting with monoclonal antibodies such as elotuzumab, daratumumab, and isatuximab. The aim of this review is summarizing the results of RRMM trials with monoclonal antibodies and of the principal ongoing trials containing them. Additionally, we put a brief focus on novel drugs (such as bispecific antibodies) to provide a better overview. The advent of monoclonal antibodies has been nothing short of a game-changer for multi-refractory patients. It has opened up a whole new world of possibilities, offering myeloma patients a brighter and more hopeful future, even in the face of relapse.

## 1. Introduction

Multiple myeloma (MM) is the second most frequent hematologic malignancy. It is characterized by an uncontrolled proliferation of monoclonal plasma cells in the bone marrow, causing destructive bone lesions, kidney injury, anemia, and hypercalcemia. MM can also affect extramedullary sites as the disease progresses. MM typically presents with a median morbidity age of 69 years, and nearly 63% of diagnosed patients are aged > 65 years [1]. Transplant-eligible patients may undergo autologous stem cell transplantation (ASCT), which deepens and prolongs the therapeutic response, improving overall outcomes. Consolidation and maintenance therapy post-ASCT further deepen the response, greatly increasing patient survival [2]. However, despite the use of triplet and quadruplet induction regimens, ASCT, and maintenance therapy, MM remains an incurable disease. Long-term treatment is required, and relapse ultimately occurs in nearly every patient with MM [2]. According to the International Myeloma Working Group (IMWG) definitions, refractory MM is defined as a disease that is nonresponsive to primary or salvage therapy or progresses within 60 days of the last therapy. There are two categories of refractory MM: relapsed-and-refractory myeloma and primary refractory myeloma. Relapsed and refractory myeloma is defined as disease that is nonresponsive to salvage therapy or progresses within 60 days of the last therapy in patients who had achieved minimal response (MR) or better at some point before progressing in their disease course [3]. When relapse occurs, its management can be complex and challenging due to the biological heterogeneity of the disease. It must take into account patient-related factors such as age, performance status, frailty, comorbidities, logistics, and preferences, as well as disease-related factors like high-risk features. Therapeutic history also plays a crucial role in selecting the best therapeutic option. Based on IMWG criteria, therapy is recommended for patients with clinical relapse. In patients who do not exhibit clinical relapse, significant paraprotein relapse is defined as a doubling of the monoclonal component (M protein), which is a key biomarker produced by malignant plasma cells. This increase should occur in two consecutive measurements separated by less than or equal to 2 months, or an increase in the absolute levels of serum M protein by more than or equal to 1 g/dL, or urine M protein by more than or equal to 500 mg/24 h, or involved FLC level by more than or equal to 20 mg/dL (plus an abnormal FLC ratio) in two consecutive measurements separated by less than or equal to 2 months. This definition of paraprotein relapse represents the rate of rise or absolute level of increase in M protein at which the panel considers that myeloma therapy should be restarted in relapsing patients in clinical practice, even if signs and symptoms of new organ damage are not yet evident [3]. In this review, the role of monoclonal antibodies in the context of relapsed multiple myeloma (MM) is discussed, with the aim of providing an overview of these therapies that have had a significant impact on the treatment landscape in this setting.

## 2. Monoclonal Antibodies in Relapsed Refractory Multiple Myeloma

### 2.1. Elotuzumab

Elotuzumab is a humanized monoclonal antibody that is specifically for signaling lymphocytic activation molecule F7 (SLAMF7). This monoclonal antibody exerts its effect on MM cells by enhancing natural killer (NK) cell-induced antibody-dependent cell cytotoxicity, NK cell activation, and antibody-dependent cell phagocytosis through macrophages [4]. Elotuzumab has been approved for use in combination with lenalidomide and dexamethasone (Elo-Rd) and pomalidomide and dexamethasone (Elo-Pd) for the treatment of relapsed myeloma patients.

- Phase I clinical trials

In the first-in-human phase I study, 35 patients with RRMM received elotuzumab as single agent in a dose escalation plan; unfortunately, no meaningful response was achieved, and most patients had a progressive disease while on treatment [5]. In another phase I study, elotuzumab was given to 28 patients with RRMM in dose escalation and in association with bortezomib; the maximum tolerated dose (MTD) was not reached in this trial and the overall response rate (ORR) was 48% [6]. Elotuzumab was also examined in a phase I clinical trial in combination with lenalidomide and dexamethasone in 29 patients with relapsed or refractory multiple myeloma (MM), including lenalidomide exposed patients (after a required period of wash-out). The ORR was 82%. The response rate among lenalidomide-exposed patients was 33%. This suggests that the combination of elotuzumab, lenalidomide, and dexamethasone may also be effective in patients previously exposed to lenalidomide; therefore, elotuzumab appeared to act in synergism with lenalidomide and dexamethasone in RRMM and this combination was elected for further clinical trials [7].

- Phase II clinical trials

A multicenter randomized phase II study tested elotuzumab in combination with a proteasome inhibitor (PI), bortezomib. In this trial 152 RRMM patients who underwent no more than three prior lines of therapy received elotuzumab with bortezomib and dexamethasone (EloVd) or bortezomib and dexamethasone (Vd) until disease progression/unacceptable toxicity. The primary endpoint was progression-free survival (PFS); secondary/exploratory endpoints included ORR and overall survival (OS). The study achieved its primary endpoint, as PFS was significantly longer with EloVd compared to Vd (9.7 vs. 6.9 mo.). Moreover, the analysis suggests that patients in the EloVd group who were homozygous for the high-affinity FcγRIIIa V allele appeared to show longer PFS compared with patients homozygous for the low-affinity allele. The FcγRIIIa receptor is expressed on natural killer cells and binds to the Fc part of elotuzumab to induce antibody-dependent cellular cytotoxicity (ADCC) [8]. In a randomized, multicenter, open-label dose-escalation phase II study 73 patients with RRMM exposed to one to three previous therapies but no previous lenalidomide were assigned (1:1) to either 10 mg/kg or 20 mg/kg intravenous elotuzumab plus oral lenalidomide (25 mg) and dexamethasone (40 mg). The ORR was 84%, higher in the 10 mg/kg cohort compared with the 20 mg/kg cohort (92% vs. 76%, respectively). After a median follow up after 18 months, the 10 mg/kg cohort had a longer PFS as compared with the higher dose cohort. The number of prior lines of therapy did not impact the response [9]. In view of these encouraging results, two phase III trials were launched, ELOQUENT-1 and ELOQUENT-2, comparing lenalidomide and low dose dexamethasone (Rd) with or without elotuzumab in patients with newly diagnosed MM and RRMM, respectively.

Considering the effectiveness of elotuzumab and the efficacy of the pomalidomide-dexamethasone combination in lenalidomide-refractory patients [10], patients with RRMM and who were refractory to lenalidomide and to a proteasome inhibitor (PI) were randomly assigned to receive elotuzumab plus pomalidomide and dexamethasone or pomalidomide and dexamethasone alone in the phase II trial ELOQUENT-3. After limited follow-up (9.1 months), the median PFS was 10.3 months in the elotuzumab group compared to 4.7 months in the control group. The ORR was 53% in the elotuzumab group, compared to 26% in the control group [11]. In the final analysis of OS from ELOQUENT-3 trial, median OS was significantly improved with EloPd (29.8 months) versus Pd (17.4 months). The risk of death was reduced by 41% with EloPd vs. Pd. In addition, the safety profile of EloPd was consistent with previous reports and the triplet combination was shown to offer substantial clinical benefits without significant toxicities. Moreover, exploratory subgroup analysis suggested improved OS with EloPd in subgroups generally associated with poor outcomes, including patients older than 75 years, patients lenalidomide and PI refractory, multi-treated patients who received at least 4 lines of prior systemic therapy and patients who had received Lenalidomide as their most recent prior line of therapy [12]. In 2018, the EloPd combination received FDA approval for RRMM patients who had undergone at least two prior lines of therapy. Recently, Gentile et al. published data from a real-world study on EloPd, administered to 200 RRMM patients across 35 Italian centers outside the context of clinical trials. The ORR was 55.4%, a finding in line with trial results. The median PFS was 7 months, which was shorter than in ELOQUENT-3, probably due to differences in the two cohorts. The median OS was shorter than that observed in the ELOQUENT 3 trial (17.5 vs. 29.8 months), but this result should be related to the relatively short follow up. These findings confirmed that EloPd is a possible therapeutic choice for RRMM patients who received at least two prior lines of therapies, including lenalidomide and a PI [13]. Based on the results obtained in the OPTIMISMM study, which showed improved outcomes with the combination of pomalidomide, bortezomib, and dexamethasone (PVd) in RRMM, a phase II trial investigated adding elotuzumab to PVd (EloPVd) in the same setting. The primary objective was to assess the ORR. Eligible patients had relapsed/refractory disease with at least one prior treatment, including lenalidomide and a PI. The trial enrolled patients with a median of 3 prior treatments. One-quarter had high-risk FISH, and prior therapies included pomalidomide (33%), daratumumab (25%), and isatuximab (4%). The best ORR was 56.3%, with a median PFS of 10 months. In patients with one prior line of therapy, the ORR was 73.7%, and median PFS was 23.4 months. In consideration of these data, EloPVd demonstrated efficacy in triple-class-exposed patients with prior anti-CD38 monoclonal antibody use [14].

- Phase III clinical trials

ELOQUENT-2 is a randomized, multicenter, phase III clinical trial which compared Elo-Rd vs. Rd in 646 RRMM patients after one to three prior lines of therapy. Prior lenalidomide exposure was permitted by the study protocol, but for enrollment the patients must not be lenalidomide refractory. Patients receiving elotuzumab had a relative reduction of 30% in the risk of disease progression or death as compared with the control group [15]. After a 4-year follow-up, Elo-Rd showed a clinically relevant improvement in PFS, in comparison with Rd and also a significant improvement in OS, after a minimum follow up of 70.6 months (48.3 months vs. 39.6 months) [16]. In addition, Elo-Rd had an acceptable safety profile and did not add hematological or non-hematological toxicity to Rd. Moreover, OS benefit was maintained among patients with adverse prognostic factors, including older age, ISS stage III, IMWG high-risk disease, and 2–3 prior lines of therapy. Therefore, EloRd was the first antibody-based triplet regimen that significantly prolonged OS in patients with RRMM [17]. Gentile et al. reported data from an Italian real-world experience with Elo-Rd as a treatment for 300 RRMM patients treated outside of controlled clinical trials, 41% of whom were aged 75 years or older. The results of this retrospective analysis were consistent with the ELOQUENT-2 trial, with an ORR of 77% and a median PFS of 17.6 months [18].

- Ongoing Clinical trials with elotuzumab in RRMM patients (compare ClinicalTrials.gov)

1. A phase I/II, open-label, non-randomized, single-stage study (NCT06518551) is going to evaluate the efficacy of elotuzumab and iberdomide therapy post-idecabtagene vicleucel in participants with RRMM. Iberdomide, a type of cereblon E3 ligase modulator, has demonstrated some antitumor activity in laboratory studies. It is expected about 49 people will take part in this research study. 

2. IMPEDE (NCT04835129) is a multicenter, open-label phase II study in subjects with relapsed and/or refractory multiple myeloma with at least two prior lines of therapy. The study’s hypothesis is that the isatuximab in combination with elotuzumab, pomalidomide, and dexamethasone (Isa-EPD) will be safe and lead to superior response rates than seen with either isatuximab with pomalidomide and dexamethasone or elotuzumab with pomalidomide and dexamethasone in subjects with relapsed and/or refractory multiple myeloma. 

3. A phase Ib trial (NCT05981209) tests the safety, side effects, and best dose of CC-92480 (medigzomide, a molecule that works by binding to a protein called CRBN that triggers the breakdown of proteins Ikaros and Aiolos, leading to cell death in multiple myeloma cells) in combination with elotuzumab and dexamethasone in treating patients with RRMM. 

Since more and more patients with RRMM are exposed to several lines of therapy, including CD38- and B-cell maturating antigen (BCMA)-targeted therapies (antibody and plasma cell treatments that help the body’s immune system to kill cancer cells) and will often relapse and become refractory to these therapies, it is important to identify effective treatment options in this setting. Elotuzumab would be considered for RRMM, having a different mechanism of action, even in association with novel molecules, like mezigdomide or iberdomide.

These ongoing clinical trials are summarized in Table 1. 

### 2.2. Daratumumab

Daratumumab is a humanized IgG1k monoclonal antibody that targets the CD38 epitope, which is a transmembrane glycoprotein that is expressed on multiple immune system cell types and in hematological malignancies, including MM. This monoclonal antibody has demonstrated its killing activity against CD38-expressing tumor cells, inducing antibody dependent cellular cytotoxicity (ADCC), complement–dependent cytotoxicity (CDC), macrophage-mediated phagocytosis and cell lysis in CD38+ myeloid derived suppressor cells and Tregs [19,20].

- Daratumumab monotherapy

There are two studies in which daratumumab showed its single-agent anti-MM activity: the phase I-II GEN501 and the phase II SIRIUS. In part 1 of GEN501, patients with RRMM received daratumumab at different doses (dose-escalation phase) without identification of a maximum tolerated dose, whereas in part 2, the cohort treated with daratumumab at 16 mg/kg achieved an ORR of 36% [21]. In phase II SIRIUS study patients with RRMM who were previously treated with at least three lines of therapy (median of 5) received daratumumab 16 mg/kg per week for 8 weeks (cycles 1 and 2), then every 2 weeks for 16 weeks (cycles 3–6), and then every 4 weeks thereafter (cycle 7 and higher). The ORR was 29% and after a median follow up of 9.3 months, median PFS was 3.7 months [22]. An updated analysis of these two studies showed an ORR of 31% and a deep and durable response; median overall survival was 20.1 months. Benefit was also shown in patients who achieved minimal response/stable disease [23]. The pooled, post hoc final analysis of these two studies showed, after a median follow up 36.6 months, an overall response rate of 30.4%. The median duration of response was 8.0 months. Median overall survival was 20.5 months, with a 3-year overall survival rate of 36.5%. Moreover, there were no new safety concerns with longer follow-up [24].

A retrospective study was conducted in a real-world setting to examine the effectiveness of daratumumab as a single agent in 41 patients with RRMM who had received at least three prior lines of therapy. Most patients exhibited double refractory disease and were refractory to their most recent treatment. While the overall response rate was comparable to that observed in previous studies, the median progression-free survival (PFS) and OS were inferior in this study. The patients included in this real-life study exhibited a higher mean age and lower functional status compared to those in previous clinical trials. Additionally, the study revealed that daratumumab exhibited a favorable safety profile, with a reduced incidence of infusion-related reactions compared to that observed in clinical studies. Furthermore, these findings emphasize the value of real-world data in providing a complementary perspective to that offered by clinical trials [25].

- Daratumumab and IMIDs

A phase 1/2 (GEN503) study was conducted to assess the combination of daratumumab with lenalidomide/dexamethasone in patients with RRMM. Part 1 of the study focused on dose escalation, determining that 16 mg/kg was the recommended phase 2 dose (RP2D) for daratumumab. Part 2 involved dose expansion at the RP2D. The combination therapy was well tolerated with manageable adverse effects, including grade 3 to 4 adverse events and infusion-related reactions. The overall response rate was 81%, with encouraging progression-free and overall survival rates [26]. The final results of this study showed that the ORR remained unchanged, but more patients achieved complete response (CR) or stringent complete response (sCR). The duration of response was not reached, with the median PFS and OS not estimable [27]. In the phase III POLLUX study, daratumumab in combination with lenalidomide/dexamethasone significantly decreased the risk of disease progression or death by 63% and increased the ORR compared to lenalidomide/dexamethasone alone in patients with RRM with at least one line of prior therapy. After more than 3 years of follow-up, D-Rd prolonged PFS, increased OSS, and led to deeper responses, including significantly higher (>5-fold) rates of MRD negativity (10–5) versus Rd alone (30.4 vs. 5.3%, respectively), which deepened with longer follow-up [28].

In the overall survival analysis of POLLUX study, after a median follow up of 79.7 months, significant OS benefit was observed in D-Rd; the median OS was 67.6 months for D-Rd vs. 51.8 months for Rd. Moreover, prespecified analysis demonstrated an improved OS with D-Rd vs. Rd in most subgroups, including patients aged 65 years and patients with one, two, or three prior lines of therapy, International Staging System stage III disease, high-risk cytogenetic abnormalities, and refractoriness to their last prior line of therapy or a PI [29].

The combination of daratumumab, pomalidomide, and dexamethasone (DPd) was evaluated in the phase 1b EQUULEUS (MMY1001) study, which assessed the safety and tolerability of daratumumab in combination with various treatment regimens. This study shows that daratumumab can be safely combined with pomalidomide plus dexamethasone (Pd). Although there were higher rates of neutropenia, the safety profiles of daratumumab plus Pd and Pd alone were similar. The addition of daratumumab to pomalidomide resulted in favorable responses in heavily treated patients with RRMM (median of 4 prior therapies). The patient population in this study was like previous studies, and the addition of daratumumab to Pdled to a higher ORR (60%), even in high-risk patients. After a median follow-up of 13.1 months, the median PFS was 8.8 months, and the median OS was 17.5 months. The study also showed promising outcomes in patients with deep and durable responses, including minimal residual disease negativity [30]. The safety and efficacy of pomalidomide, low-dose dexamethasone, and daratumumab were evaluated in lenalidomide-pretreated patients with RRMM in the phase 2 MM-014 study. The study included 112 patients, all of whom had received lenalidomide in their most recent prior regimen. The ORR was 77.7% and the median PFS was not reached, with a 1-year PFS rate of 75.1%. The most common grade 3/4 treatment-emergent adverse event was neutropenia. These results demonstrate the safety and efficacy of pomalidomide-based therapy as early as second line in patients with RRMM, even after lenalidomide failure, suggesting that switching from the immunomodulatory agent class is not necessary [31]. Moreover, Piercell at al. showed that patients treated with DPd did not experience dysfunction in their innate and adaptive immune systems. Instead, they exhibited noticeable growth in CD4, CD8, and NK cell subsets after treatment. Previous research had indicated that pomalidomide can boost T cell- and NK cell-driven immune responses, suggesting that its administration may help counteract the immunosuppressive effects of daratumumab and dexamethasone [32]. The immune response seen in patients treated with pomalidomide after relapsing or becoming refractory to lenalidomide suggests that pomalidomide has distinct pharmacological properties and remains effective in cases of lenalidomide resistance [33]. In the phase III trial APOLLO, 304 patients with RRMM who have undergone at least one treatment regimen were randomly assigned to the DPd (with subcutaneous daratumumab) or the Pd group. At a median follow up of 16.9 months, the DPd group showed improved PFS compared to the control group (12.4 vs. 6.9 months). The most common grade 3–4 adverse event was neutropenia (68% vs. 51% of patients). Patients receiving DPd achieved faster and deeper responses with longer duration that those of the control group and had a reduced risk of disease progression or death [34].

- Daratumumab and PI

The phase III trial CASTOR evaluated bortezomib and dexamethasone (Vd) with or without daratumumab (DVd). In this study, 498 patients with RRMM who were previously exposed to a median of two therapies were randomly assigned to receive bortezomib and dexamethasone alone or in combination with daratumumab. Approximately half of the patients were treated with PIs and IMIDs [35]. After three years of follow up, median PFS was significantly prolonged with DVd versus Vd (16.7 vs. 7.1 months). In lenalidomide-refractory patients, the median PFS was 7.8 versus 4.9 months for DVd vs. Vd. Minimal residual disease (MRD) negativity rates (10^−5^) were greater with DVd versus Vd [36]. Post hoc analyses of CASTOR and POLLUX evaluated patient subgroups based on timing of progression/relapse (early or late) after first line of therapy. PFS favored the daratumumab-containing regimens across subgroups using both a 24- and 18-month early-relapse cutoff. In the CASTOR/POLLUX pooled data set, daratumumab reduced the risk of disease progression or death by 65% in the early-relapse (<24 months) subgroup and by 65% in the late-relapse (≥24 months) subgroup. OS also favored the daratumumab-containing regimens in both subgroups. Although daratumumab is unable to fully overcome the adverse prognosis of early relapse, these results support the use of daratumumab for patients with 1 prior line of therapy, including for those who progress/relapse early after initial therapy and are considered to have functional high-risk MM (defined as patients with no markers of the genomic high-risk group but who were refractory to induction therapy or had early relapse within 12 months) [37]. The phase 1b EQUULEUS study (MMY1001), besides DPd, assessed the validity of the association of daratumumab, carfilzomib, and dexamethasone in 85 patients with RRMM [38]. The efficacy of this combination was confirmed in the phase III CANDOR study, where 466 RRMM patients were randomized to receive either Kd (carfilzomib on days 1, 2, 8, 9, 15, 16 at a dose of 20 mg/m^2^ on days 1 and 2 of cycle 1, and 56 mg/m^2^ thereafter; dexamethasone 40 mg weekly) or KdD (Kd plus daratumumab 16 mg/kg with the same schedule as the MMY1001 study). The final analysis of this study, after a median follow up period of 50 months, showed that patients treated with KdD had higher minimal residual disease–negative (MRD−) achievement rates (28% vs. 9%) than those treated with Kd. Median PFS was 28.4 months for KdD vs. 15.2 months for Kd. The median OS for KdD was 50.8 months vs. 43.6 months for Kd. Improvements in overall survival trends were observed across predefined subgroups, including patients refractory to lenalidomide and those refractory to PI. A significant improvement was noted in patients with high-risk cytogenetics (KdD: 34.3 months vs. Kd: 17.1 months) [39]. A single-arm phase 2 study (NCT03439293) evaluated ixazomib combined with daratumumab and dexamethasone (IDd) in 61 RRMM patients (ixazomib/daratumumab-naïve; 1–3 prior therapies), who received a median of 16 IDd cycles. The final analysis showed that the ORR was 64.4%, with a confirmed ≥very good partial response (VGPR) rate of 30.5%, including 26.7% in high-risk cytogenetics and 28.6% in lenalidomide-refractory patients. The median PFS was 16.8 months, with a median follow-up of 31.6 months. Grade ≥3 treatment-emergent adverse events (TEAEs) occurred in 54.1%, leading to dose modifications (62.3%) or discontinuations (16.4%). Peripheral neuropathy was reported in 18% (any grade) and 1.6% (grade ≥3). Quality of life was maintained, and IDd demonstrated efficacy and a favorable safety profile, including in high-risk subgroups [40].

- Daratumumab plus venetoclax

A phase I study (NCT03314181) evaluated venetoclax with daratumumab and dexamethasone (VenDd) in RRMM patients with t(11;14) translocation, and venetoclax with daratumumab, dexamethasone, and bortezomib (VenDVd) in cytogenetically unselected RRMM patients. Among 48 enrolled patients (24 in each cohort), the ORR was 96% for VenDd. The 18-month PFS rate was 90.5% with VenDd and 66.7% with VenDVd. Common adverse events included diarrhea (63% VenDd, 54% VenDVd) and nausea (50% in both groups). Grade ≥3 adverse events occurred in 88% of VenDd and 71% of VenDVd patients. VenDd and VenDVd demonstrated deep, durable responses with manageable safety profiles, supporting further evaluation of venetoclax-based regimens, especially for patients with t(11;14) [41].

- Daratumumab plus Selinexor

A phase II study (SELIBORDARA, NCT03589222) evaluated selinexor, an exportin-1 inhibitor, combined with daratumumab, bortezomib, and dexamethasone (S-DVd) in RRMM. Fifty-seven patients were enrolled: 24 in part 1 (heavily pretreated, median of three prior lines [PLs]) and 33 in part 2 (early relapse, median of one PL). In part 1, the overall ORR was 50%, with a median PFS of 7 months. In part 2, the ORR was 82%, with CR or better in 33% and a median PFS of 24 months, including 22.1 months in lenalidomide-refractory patients. Thrombocytopenia and nausea were the most common adverse events, with 62% of patients requiring dose modifications. While the primary endpoint was not met, S-DVd demonstrated promising efficacy and a manageable safety profile, warranting further investigation as a potential treatment option for RRMM [42].

- Subcutaneous daratumumab

The phase III COLUMBA trial evaluated the non-inferiority of subcutaneous (SC) versus intravenous daratumumab in RRMM patients. Eligible adults (≥18 years) had received ≥3 prior therapy lines, including a PI and immunomodulatory drug, or were double refractory, with an ECOG performance status ≤2. Patients were randomized 1:1 to receive either 1800 mg SC daratumumab co-formulated with recombinant hyaluronidase or 16 mg/kg IV daratumumab on the same schedule: weekly (cycles 1–2), biweekly (cycles 3–6), then every 4 weeks. Co-primary endpoints were ORR and maximum trough concentration on cycle 3, day 1. Non-inferiority for ORR was based on retaining 60% of the lower bound (20.8%) from the SIRIUS trial [43]. The Phase II PLEIADES study evaluated subcutaneous daratumumab (DARA SC) with standard-of-care regimens for MM: DVRd (bortezomib/lenalidomide/dexamethasone) in transplant-eligible newly diagnosed MM (NDMM), D-VMP (bortezomib/melphalan/prednisone) in transplant-ineligible NDMM, and DRd (lenalidomide/dexamethasone) in RRMM. Among 199 patients (DVRd: 67, D-VMP: 67, D-Rd: 65), primary endpoints were met: ≥VGPR after four 21-day cycles for DVRd was 71.6%, and ORRs were 88.1% for DVMP and 90.8% for D-Rd. With follow-up (DVMP: 14.3 months, D-Rd: 14.7 months), responses deepened (ORRs: 89.6%, 93.8%; ≥VGPR: 77.6%, 78.5%) with MRD negativity rates of 16.4% and 15.4%. Infusion-related reactions were mild and infrequent (≤9.0%), and DARA SC administration took a median of 5 min. Therefore, DARA SC showed similar efficacy to IV formulations with fewer infusion-related reactions and shorter administration time [44].

- Daratumumab plus melflufen

In the phase I/II ANCHOR study, the combination of melphalan flufenamide (melflufen), a first-in-class alkylating peptide-drug conjugate, 30 or 40 mg, plus daratumumab and dexamethasone demonstrated clinical activity and manageable safety in patients RRMM refractory to (or intolerant of) an IMiD and/or PI, with 1–4 prior lines of therapy [45]. Melflufen was subsequently evaluated in the LIGHTHOUSE study (NCT04649060), comparing melflufen plus daratumumab and dexamethasone (melflufen group) to daratumumab alone in RRMM patients. The trial was prematurely closed due to a partial clinical hold by the FDA and financial constraints, with 54 of 240 planned patients randomized (27 per group). At a median follow-up of ~7 months, median PFS was not reached in the melflufen group compared to 4.9 months in the control group. ORR was 59% in the melflufen group versus 30% in the control group. Common grade ≥3 adverse events in the melflufen group included neutropenia (50%), thrombocytopenia (50%), and anemia (32%). Melflufen combined with daratumumab plus dexamethasone showed superior PFS and ORR with a safety profile consistent with prior studies, supporting its potential role in treating RRMM [46].

- Ongoing trials with daratumumab in RRMM patients (compare ClinicalTrials.gov).

1. NCT04176718, a phase II, multicenter, open-label study that is evaluating daratumumab in combination with weekly carfilzomib, pomalidomide, and dexamethasone in people with RRMM who have received at least one prior therapy and who have had previous treatment with both lenalidomide and a PI.

2. NCT05020236, MAGNETISMM 5, a randomized phase III study that aims to evaluate the BCMA-CD3 bispecific antibody elranatamab, alone or in combination with daratumumab, compared to a regimen of daratumumab, pomalidomide, and dexamethasone in multiple myeloma patients previously treated with lenalidomide and a proteasome inhibitor. Part 1 assesses the safety and activity of elranatamab at different doses combined with daratumumab. In Part 2, participants will be randomized to receive elranatamab alone, elranatamab plus daratumumab, or the daratumumab-pomalidomide-dexamethasone regimen. The study will compare the safety and efficacy of elranatamab alone and in combination with daratumumab against the standard regimen.

3. NCT05455320, a phase III randomized study that aims to compare the efficacy of subcutaneous (SC) talquetamab combined with daratumumab and pomalidomide (Tal-DP) and talquetamab combined with daratumumab (Tal-D) to the combination of daratumumab, pomalidomide, and dexamethasone (DPd).

4. NCT04975997, a multicenter two-stage, randomized, controlled Phase III study that aims to compare the efficacy and safety of iberdomide in combination with dexamethasone and daratumumab (IberDd) versus daratumumab, bortezomib, and dexamethasone (DVd) in participants with RRMM.

5. NCT05896228, a phase 2, single-arm study of iberdomide, daratumumab, carfilzomib, and dexamethasone (IberKDd) in patients with RRMM; in this study, the investigators aim to determine whether the experimental combination of iberdomide, carfilzomib, daratumumab, and dexamethasone (IberKDd) offers better outcomes for patients with RRMM compared to current standard-of-care treatments. This study will evaluate the tolerability and efficacy of IberKDd in all participants, as well as its ability to reduce or prevent the recurrence of MM.

These ongoing clinical trials are summarized in Table 1.

### 2.3. Isatuximab

Isatuximab is an IgG1k chimeric monoclonal antibody that binds to a unique epitope on human CD38, targeting a distinct amino acid sequence different from that of the anti-CD38 monoclonal antibody daratumumab. It demonstrates unique, strong proapoptotic activity without requiring cross-linking agents and employs potent effector mechanisms, including antibody-dependent cell-mediated cytotoxicity (ADCC), complement-dependent cytotoxicity (CDC), and antibody-dependent cellular phagocytosis (ADCP), against CD38+ malignant cells. Additionally, isatuximab inhibits the ADP-ribosyl cyclase activity of CD38, likely through allosteric antagonism, activates natural killer (NK) cells even in the absence of CD38+ tumor cells, and suppresses CD38+ regulatory T cells [47].

- Isatuximab single agent

A phase I dose-escalation study assessed isatuximab monotherapy in patients with RRMM. Patients received intravenous isatuximab weekly (QW) or every two weeks (Q2W). The primary goal was to identify the maximum tolerated dose (MTD), which was not reached, and no cumulative adverse reactions were observed. CD38 receptor occupancy plateaued at 80% with the highest tested dose of 20 mg/kg, correlating with clinical responses. Among patients treated with isatuximab at doses ≥ 10 mg/kg, the ORR was 23.8%, including one complete response [48].

This findings have been validated in a phase 2 dose-finding study evaluated isatuximab, in which 97 patients with ≥3 prior therapy lines or dual refractory were randomized to different dosing regimens: 3 mg/kg Q2W, 10 mg/kg Q2W/Q4W, 10 mg/kg Q2W, or 20 mg/kg QW/Q2W.The ORR ranged from 4.3% (3 mg/kg Q2W) to 29.2% (10 mg/kg Q2W), with the highest efficacy observed at doses ≥10 mg/kg. At these doses, median PFS was 4.6 months, OS was 18.7 months [49]. After these results, a phase 2 study evaluated isatuximab as monotherapy or combined with dexamethasone (Isa-dex) in patients with the same characteristics of the prior group. A total of 164 patients were treated. The ORR was 23.9% in the monotherapy group (Isa) and 43.6% in the combination group (Isa-dex), with the addition of dexamethasone significantly improving outcomes. Median PFS was 4.9 months for Isa and 10.2 months for Isa-dex, while median OS was 18.9 months and 17.3 months, respectively. No detrimental effect on safety were observed [50].

- Isatuximab and IMIDs

In a phase 1b dose-escalation study isatuximab was tested in combination with lenalidomide and dexamethasone in patients with RRMM. Patients received isatuximab at various dosing schedules (3, 5, or 10 mg/kg Q2W; or 10 or 20 mg/kg QW/Q2W) alongside lenalidomide (25 mg, days 1–21) and dexamethasone (40 mg QW) in 28-day cycles. Among 57 patients (median 5 prior regimens, 83% refractory to lenalidomide), the median treatment duration was 36.4 weeks. The maximum tolerated dose (MTD) was not reached, with only one dose-limiting toxicity (grade 3 pneumonia) observed. Infusion-associated reactions (IARs) occurred in 56% of patients, mostly grade 1/2 and during the first infusion. The ORR was 56% across cohorts and 52% in lenalidomide-refractory patients [51].

Another Phase 1b dose-escalation study assessed isatuximab combined with pomalidomide and dexamethasone in patients with RRMM who had received ≥2 prior therapies, including lenalidomide and a PI. Patients were treated with isatuximab (5, 10, or 20 mg/kg weekly for 4 weeks, then Q2W), pomalidomide (4 mg, days 1–21), and dexamethasone (40 mg weekly) in 28-day cycles. Among 45 patients (91% refractory to their last regimen, 82% lenalidomide-refractory, 84% PI-refractory), the median treatment duration was 9.6 months, with 42% still on therapy. The most common adverse events were fatigue (62%), upper respiratory tract infection (42%), infusion reactions (42%), and dyspnea (40%). The overall response rate was 62% and a median progression-free survival of 17.6 months. The 10 mg/kg weekly/Q2W dose of isatuximab was selected for future studies, demonstrating significant clinical activity and a manageable safety profile in heavily pretreated and PI refractory patients [52]. Based on these findings, the phase 3 study ICARIA evaluated the efficacy and safety of adding isatuximab to pomalidomide and dexamethasone in patients with RRMM who had received at least two prior lines of treatment, including lenalidomide and a PI. Patients who were refractory to anti-CD38 antibodies were excluded. A total of 307 patients were randomized 1:1 to isatuximab-pomalidomide-dexamethasone (Isa-Pd) or pomalidomide-dexamethasone (Pd). At a median follow-up of 11.6 months, median PFS was significantly longer in the Isa-Pd group (11.5 months) compared to the Pd group (6.5 months). The most common adverse events in the Isa-Pd group included infusion reactions (38%), upper respiratory tract infections (28%), and diarrhea (26%). These data showed that isatuximab significantly improves PFS and offers a valuable treatment option for RRMM, particularly in patients refractory to lenalidomide and PI [53]. These findings were confirmed by the final overall survival analysis: after a median follow-up duration of 52.4 months, the median OS was 24.6 months with IsaPd and 17.7 months with Pd. Median PFS2 was significantly longer with IsaPd vs. Pd (17.5 vs. 12.9). A clinically meaningful 6.9-month improvement in median OS was obtained [54]. This advantage was preserved in all subgroups, in particular in high-risk cytogenetic MM [55], in elderly patients (aged 75 years or older) [56], in lena-refractory, PI refractory or double refractory patients [57]. Even in patients with renal impairment (RI; eGFR <60 mL/min/1.73 m^2^), IsaPd showed improved outcomes; moreover, Isa pharmacokinetics were unaffected by RI, suggesting no need for dose adjustment [58]. The superiority of IsaPd over Pd in terms of ORR, PFS, OS and depth of response was maintained in +1q patients. This cytogenetic abnormality (+1q) refers to extra copies of chromosome 1q in malignant plasma cells, with gain(1q) indicating one additional copy (three total) and amp(1q) indicating two or more additional copies (four or more total). The lack of consistent cytogenetic reporting has complicated our understanding of +1q’s role, but its negative prognostic impact is evident, particularly when combined with other high-risk abnormalities (“double-hit” or “triplet-hit” scenarios). +1q is now incorporated into updated MM staging systems, emphasizing its clinical importance [59]. Based on the results of ICARIA-MM, isatuximab is approved in several countries in combination with Pd for the treatment of adult patients with RRMM who have received ≥2 prior therapies, including lenalidomide and aPI.

Regarding real-world evidence, the IMAGE study represents a recent retrospective cohort analysis of patients with RRMM in France who were treated with Isa-Pd as part of an early access program. The study demonstrated a median progression-free survival (mPFS) of 12.4 months, which is comparable to the mPFS observed in clinical trials of anti-CD38 monoclonal antibodies in combination with pomalidomide and dexamethasone. These include the Isa-Pd arm of ICARIA-MM (mPFS 11.1 months, 95% CI 7.8–13.8) and the Dara-Pd arm of APOLLO (mPFS 12.4 months, 95% CI 8.3–19.3). The median number of prior lines of therapy (LOTs) for patients in IMAGE was two, which is comparable to the number observed in APOLLO. In contrast, ICARIA-MM included patients with a median of three prior LOTs. In contrast to the CD38-naïve populations in ICARIA-MM and APOLLO, 19.1% of IMAGE patients exhibited refractoriness to daratumumab [60].

- Isatuximab and proteasome inhibitors

The phase III IKEMA trial compared isatuximab plus carfilzomib-dexamethasone to carfilzomib-dexamethasone alone in patients with RRMM. This study enrolled 302 patients with 1–3 prior therapy lines. The isatuximab group received 10 mg/kg intravenously weekly for 4 weeks, then every 2 weeks, whereas both groups followed the approved regimens of carfilzomib and dexamethasone. The median progression-free survival (PFS) was not reached in the isatuximab group, whereas it was 19.15 months in the control group. Grade 3 or worse adverse events (TEAEs) occurred in 77% (isatuximab) vs. 67% (control). Therefore, adding isatuximab to carfilzomib-dexamethasone significantly improved progression-free survival and response depth [61]. The updated results from IKEMA showed that Isa-Kd significantly prolonged PFS compared to Kd alone (median 35.7 vs. 19.2 months). This benefit was consistent across all subgroups, including high-risk patients. Moreover, Isa-Kd improved stringent complete response and MRD negativity (33.5% vs. 15.4%). The safety profile of Isa-Kd remained consistent with the interim analysis, with manageable adverse events [62]. In a recent Italian multicenter retrospective study, the real-world efficacy and safety of IsaKd was evaluated in 103 RRMM patients. The ORR was 85%, including 18% achieving stringent/complete response (sCR/CR) and 39% very good partial response (VGPR). Median progression-free survival (PFS) and OS were not reached, but 1-year PFS and OS rates were 72% and 77%, respectively. These findings confirmed the efficacy of Isa-Kd in real-world RRMM settings, supporting its role as an effective treatment option [63]. The superiority of IsaKd regimen was confirmed in the 1q21+ patients and related subgroups in the IKEMA study, at long-term follow-up (44.2 months) [64]. In a recent subgroup analysis in patients with functional high risk in the IKEMA study, Facon et al. showed that, among patients relapsing within 12 months, Isa-Kd improved PFS (24.7 vs. 17.2 months) and achieved higher rates of minimal residual disease negativity (MRD−: 24.6% vs. 15.2%) and MRD− complete response (≥CR: 18.0% vs. 10.9%) compared to Kd. Nevertheless, Isa-Kd showed even greater benefits in patients with later relapses (median PFS: 42.7 vs. 21.9 months) with higher MRD− (37.5% vs. 16.7%) rates [65].

- Isatuximab and bendamustine

A phase Ib trial evaluated the safety and efficacy of the association of isatuximab, bendamustine plus prednisone (IsaBp) in 15 patients RRMM with triple class refractory (TCR). This regimen was designed for rapid disease control, leveraging the cytotoxicity of isatuximab and the cytoreductive properties of bendamustine. Isatuximab was administered at a dose of 10 mg/kg IV weekly (cycle 1), then every two weeks; bendamustine was administered on days 1 and 2 at escalating doses (50, 75, and 100 mg/m^2^) and prednisone at 125 mg on day 1 and 60 mg on days 2–4. The purpose was to establish the maximally tolerated dose (MTD) or recommended phase 2 dose (RP2D). The overall response rate was 20%, including one stringent complete response, and the median PFS was 2.5 months. The IsaBp regimen was safe and well-tolerated, with manageable toxicities and some efficacy even in highly refractory TCR MM patients. However, the study was discontinued due to slow accrual [66].

- Ongoing trials with isatuximab in RRMM patients (compare ClinicalTrials.gov)

1. IMPEDE trial is a phase II study that aims to evaluate safety and efficacy of isatuximab in combination with elotuzumab, pomalidomide, and dexamethasone (Isa-EPD) in patients with RRMM who have undergone at least two prior lines of therapy. The hypothesis is that Isa-EPD will be safe and demonstrate superior response rates compared to combinations of isatuximab or elotuzumab with pomalidomide and dexamethasone alone.

2. ISABELA trial is a phase 2 study that aims to evaluate the overall response rate of isatuximab, belantamab mafodotin, pomalidomide, and dexamethasone in RRMM.

3. NCT04287855 is a multicenter open label phase 2 study of isatuximab plus pomalidomide and dexamethasone with carfilzomib in relapsed or refractory multiple myeloma.

These ongoing clinical trials are summarized in Table 1. 

## 3. Antibody Drug Conjugates and Bispecific Antibodies in RRMM

We will briefly mention some of the antibody drug conjugates and bispecific antibodies that are more advanced in terms of commercialization, as they are similar in pharmacological construct to monoclonal antibodies and useful in the MMRR setting, although this is beyond the scope of this review.

### 3.1. Belantamab Mafodotin

Belantamab mafodotin is a first-in-class monoclonal antibody-drug conjugate (ADC) that combines monomethyl auristatin F (MMAF), a microtubule inhibitor, with a BCMA-targeting antibody to deliver MMAF to its therapeutic target [67]. Initially evaluated in the DREAMM-1 safety trial, it underwent dose optimization in the DREAMM-2 trial in patients with RRMM who had received at least three prior therapies. In DREAMM-2, patients receiving 2.5 mg/kg of belantamab mafodotin achieved an ORR of 32%, a PFS of 2.8 months, and an OS of 15.3 months [68]. These results led to FDA and European approval for use in patients with RRMM who had received at least four prior therapies. However, in the DREAMM-3 trial, which included patients with RRMM who had received at least two prior therapies, belantamab mafodotin failed to demonstrate significant superiority in PFS compared to the pomalidomide-dexamethasone (Pd) control regimen [69]. As the trial failed to confirm a benefit over Pd, the FDA requested the withdrawal of belantamab mafodotin from the US market and the European Medicines Agency declined to renew its marketing authorization. However, in DREAMM 7, a phase 3 trial comparing belantamab mafodotin, bortezomib, and dexamethasone (BVd) with daratumumab, bortezomib, and dexamethasone (DVd) in RRMM after at least one prior therapy, median PFS was significantly longer in the BVd group (36.6 months) compared with the DVd group (13.4 months). The OS at 18 months was 84% for BVd and 73% for DVd. Therefore, BVd therapy demonstrated a superior PFS benefit over DVd in RRMM patients, although it was associated with a higher incidence of serious adverse events, such as ocular events [70].

### 3.2. Teclistamab

Teclistamab, a bispecific antibody targeting BCMA and CD3, redirects T cells to attack multiple myeloma (MM) cells. In the phase 1 MajesTEC-1 study, teclistamab was evaluated in heavily pretreated patients with a median of six prior therapies using a graded dose regimen. The subsequent Phase 1–2 study confirmed that a once-weekly subcutaneous dose of teclistamab (1.5 mg/kg after tapering) induced deep and durable responses in patients with triple-classified RRMM, consistent with previous findings [71]. Teclistamab was approved by the FDA in October 2002 based on the results of the MajesTEC-1 trial [72]. While teclistamab has shown promising therapeutic effects, further long-term studies are needed to assess its efficacy in the context of soluble BCMA levels, which may interfere with BCMA-targeted treatments, and to address the potential for relapse due to BCMA shedding after treatment [73,74].

### 3.3. Talquetamab

Talquetamab, a bispecific antibody targeting GPRC5D and CD3, induces CD3+ T cells to attack GPRC5D+ MM cells. Preclinical studies have shown its ability to induce cytolysis of GPRC5D+ MM cells without significant toxicity, even in heavily treated RRMM patients, including those resistant to anti-CD38 or anti-BCMA therapies [75]. MonumenTAL-1, a phase 1/2 study, enrolled 288 RRMM patients who received talquetamab at doses of 0.4 mg/kg weekly or 0.8 mg/kg every other week. More than 30% of participants were penta-refractory, but both dose groups achieved an ORR of more than 70%. Common grade 3/4 treatment-emergent adverse events included dose-related hematological toxicity, skin-related adverse events, and nail/taste changes affecting 60% and 50% of patients, respectively [74,76]. Despite the promising results, relapses associated with loss of GPRC5D or the emergence of drug-resistant subclones have been observed. Nevertheless, GPRC5D remains a valuable target for further research.

### 3.4. Elranatamab

Elranatamab is a bispecific antibody targeting CD3 and BCMA, which activates cytotoxic T-lymphocyte responses against BCMA-expressing myeloma cells [77]. In the Phase 2 MagnetisMM-3 trial, subcutaneous ELRA monotherapy demonstrated deep and durable responses in patients with BCMA-naive RRMM. This long-term analysis, conducted 26 months after the last patient started treatment, includes results following the switch to once-every-four-weeks (Q4W) dosing. The study enrolled heavily pretreated patients (median of 5 prior therapies) and achieved a confirmed ORR of 61%, with 37.4% achieving a complete response or better. The median duration of response and progression-free survival was 17.2 months, while overall survival was 24.6 months. Among patients who switched to Q4W dosing, 92% maintained their response, including 84% with a complete response or better. Safety profiles showed a reduction in hematological and respiratory adverse events following the switch to Q4W dosing. These results confirm the long-term efficacy and safety of ELRA and suggest that reduced dosing frequency may improve tolerability without compromising efficacy [78].

## 4. Discussion 

In the context of proteasome inhibitors (PIs) and immunomodulatory drugs as the cornerstone of treatment, the CD38 monoclonal antibodies daratumumab and isatuximab continue to play a leading role at the forefront of multiple myeloma therapy. Triplet regimens containing these monoclonal antibodies have been shown to be superior to two-drug combinations in terms of ORR, PFS, and OS in all studies, and to induce deeper responses more rapidly, even in subgroup analyses. Elotuzumab, a monoclonal antibody with an emerging target (SLAMF7) in addition to the PVD triplet, has shown efficacy in RRMM, even in patients previously exposed to anti-CD38 antibodies. However, in addition to monoclonal antibodies, other classes of immunotherapeutic drugs are emerging, such as belantamab mafodotin, which has regained a role in combination regimens for the treatment of RRMM following evidence from the DREAMM-7 study, or bispecific antibodies, such as those targeting CD3 and BCMMA or GPRC5D, which show impressive results in the setting of refractory relapsed patients. However, emerging immunotherapeutic strategies, such as CAR T-cell therapies, are expected to play a pivotal role in improving outcomes for patients with RRMM. We have provided an overview of this current topic, acknowledging the potential limitation of bias in the selection and interpretation of the studies. 

## 5. Conclusions

Monoclonal antibodies targeting CD38, such as daratumumab and isatuximab, have become central to the treatment of RRMM, proving to be more effective than two-drug combinations in terms of overall response rate, progression-free survival, and overall survival (as highlighted in Table 2)The addition of elotuzumab to triplet regimens further enhances outcomes, with the potential to improve survival rates and quality of life for patients in the refractory relapsed setting. Beyond these well-established monoclonal antibodies, novel immunotherapeutic agents, such as ADC and bispecific antibodies, or CAR T-cell therapies are emerging as promising options, with the potential to offer substantial benefits for RRMM patients. As these novel therapies continue to evolve, they are expected to reshape the treatment landscape for RRMM, providing clinicians with more targeted and effective options to manage this challenging disease.

## Figures and Tables

**Table 1:** Ongoing clinical trials with monoclonal antibodies in RRMM.

Study	Phase	NCT Identifier
Elotuzumab and iberdomide therapy post-idecabtagene vicleucel	Phase I/II	NCT06518551
Isatuximab, pomalidomide, elotuzumab and dexamethasone (IMPEDE)	Phase II	NCT04835129
Elotuzumab, CC-92480, and dexamethasone for the treatment of RRMM after CD38- and BCMA-targeted therapies	Phase Ib	NCT05981209
Daratumumab, carfilzomib, pomalidomide, dexamethasone	Phase II	NCT04176718
Study of elranatamab (PF-06863135) monotherapy and elranatamab + daratumumab versus daratumumab + pomalidomide + dexamethasone in participants with RRMM (MAGNETISMM-5)	Phase III	NCT05020236
A study comparing talquetamab in combination with daratumumab or in combination with daratumumab and pomalidomide versus daratumumab in combination with pomalidomide and dexamethasone in participants with RRMM (MonumenTAL-3)	Phase III	NCT05455320
Open-label study comparing iberdomide, daratumumab and dexamethasone (IberDd) versus daratumumab, bortezomib, and dexamethasone (DVd) in participants RRMM (EXCALIBER-RRMM)	Phase III	NCT04975997
Iberdomide, daratumumab, carfilzomib, and dexamethasone (Iber-KDd) in patients with RRMM (Iber-KDd)	Phase III	NCT05896228
Isatuximab, belantamab mafodotin, pomalidomide, and dexamethasone in RRMM (ISABELA)	Phase II	NCT05922501
Multicenter open label phase 2 study of isatuximab plus pomalidomide and dexamethasone with carfilzomib in RRMM	Phase II	NCT04287855

**Table 2:** Main clinical trials for RRMM.

Class	Name	Study	Treatment	Phase	Number of Patients	Prior LOTs	Median PFS (Months)	Median OS (Months)
Monoclonal antibodies (mAbs)	Elotuzumab	ELOQUENT 2	EloRd vs. Rd	III	646 (321 vs. 325)	1–3	19.4 vs. 14.9	48.3 vs. 39.6
	Elotuzumab	ELOQUENT 3	EloPd vs. Pd	II	117 (60 vs. 57)	≥2	10.3 vs. 4.7	28.8 vs. 17.4
	Daratumumab	POLLUX	DRd vs. Rd	III	569 (286 vs. 283)	≥1	-	67.6 vs. 51.8
	Daratumumab	EQUULEUS	DPd	Ib	103	≥2	8.8	17.5
	Daratumumab	APOLLO	DPd vs. Pd	III	304 (151 vs. 153)	≥1	12.4 vs. 6.9	-
	Daratumumab	CASTOR	DVd vs. Vd	III	498 (251 vs. 247)	≥1	16.7 vs. 7.1	49.6 vs. 38.5
	Daratumumab	CANDOR	DKd vs. Kd	III	466 (312 vs. 154)	1–3	28.4 vs. 15.2	50.8 vs. 43.6
	Isatuximab	ICARIA-MM	IsaPd vs. Pd	III	307 (154 vs. 153)	≥2	11.5 vs. 6.5	24.6 vs. 17.7
	Isatuximab	IKEMA	IsaKd vs. Kd	III	302 (179 vs. 123)	1–3	35.7 vs. 19.2	-
Antibody-drug conjugate (ADC)	Belantamab	DREAMM2	Belantamab 2.5 mg/kg vs. Belantamab 3.4 mg/kg	II	196 (97 vs. 99)	≥3	2.8 vs. 3.9	15.3 vs. 14
		DREAMM7	BVd vs. DaraVd	III	494 (243 vs. 251)	≥1	36.6 vs. 13.4	-
Bispecific antibodies	Teclistamab	MajesTEC-1	Teclistamab 1.5 mg/kg	I/II	165	≥3	11.3	18.3
	Talquetamab	MonumenTAL-1	Talquetamab 0.4 mg/kg vs. Talquetamab 0.8 mg/kg	I/II	288 (143 vs. 145)	≥3	7.5 vs. 11.9	-
	Elranatamab	MagnetisMM 3	Elranatamab 76 mg	II	123	≥3	17.2	24.6

Abbreviations: D, daratumumab; d, dehamethasone; Elo, elotuzumab; Isa, Isatuximab; K, carfilzomib, R, lenalidomide, V, bortezomib, LOTs, lines of therapies; N, number of recruiting people; PFS, progression-free survival; OS, overall survival.

## Data Availability

The data presented in the study are openly available.
